# Molecular detection of sub-microscopic infections and *Plasmodium falciparum histidine-rich protein-2* and *3* gene deletions in pre-elimination settings of South Africa

**DOI:** 10.1038/s41598-024-60007-8

**Published:** 2024-07-11

**Authors:** Olukunle O. Oyegoke, Olusegun P. Akoniyon, Leah Maharaj, Taiye S. Adewumi, Samson A. Malgwi, Samuel A. Aderoju, Abiodun J. Fatoba, Matthew A. Adeleke, Rajendra Maharaj, Moses Okpeku

**Affiliations:** 1 Discipline of Genetics, School of Life Sciences, University of KwaZulu-Natal, Durban, South Africa; 2 Department of Molecular Biosciences, University of Kansas, Lawrence, KS 66046 USA; 3 Department of Mathematics and Statistics, Kwara State University, Ilorin, Nigeria; 4 Malaria Research Unit, South African Medical Research Council, Durban, South Africa; 5 Department of Genetics, Genomics and Bioinformatics, University of Tennessee Health Science Centre, Memphis, TN 38016 USA

**Keywords:** Genetics, Microbiology

## Abstract

South Africa’s efforts toward eliminating malaria have positioned the country in the pre-elimination stage. Imported and sub-microscopic cases still contribute to the persistence of malaria in regions of low transmission as identified in this study where diagnostics is built largely on the use of Rapid Diagnostic Test (RDT). However, the presence of *Pfhrp2/3* gene deletion is known to interfere with the accuracy of diagnosis with the use of RDT. Malaria elimination and detection of *Pfhrp2/3* gene deletion in the pre-elimination setting requires accurate molecular surveillance. With the core objective of this study being the determination of the presence sub-microscopic malaria cases and deleted *Pfhrp2/3* gene markers, a total of 354 samples were collected from five districts of KwaZulu Natal, South Africa. These samples were prepared for molecular analysis using primers and PCR conditions specific for amplification of *18S rRNA* and *msp-1*gene. Positive amplicons were analysed for the presence of *Pfhrp2/3* and flanking genes, along with Sanger sequencing and phylogenetic studies. Out of 354 samples collected 339 were tested negative with PfHRP2 based RDTs. Of these *Pfhrp2* and *Pfhrp3* gene deletions were confirmed in 94.7% (18/19) and 100% (19/19) respectively. High migration rate (75%) among the study participants was noted and phylogenetic analysis of sequenced isolates showed close evolutionary relatedness with India, United Kingdom, Iran, and Myanmar and China isolates. Molecular-based test is recommended as an essential surveillance tool for malaria management programs as the target focuses on elimination.

## Introduction

The race to eliminating malaria has gained momentum in recent times and received particular attention from many malaria endemic areas across the globe. Despite all the efforts, the challenges are far from over^1,2^.

As a country whose goal is to eliminate malaria, South Africa has been working in recent times to attain this. Various control measures introduced in accomplishing this include the widespread application of insecticides, which thus far has positioned the country in the pre-elimination stage^3,4^. Northern KwaZulu Natal, Limpopo, and Mpumalanga are the three provinces in the country that still record local malaria cases with rate of 0.1, 1–0.1 and > 1 per 1000 population at risk respectively^4,5^. Imported malaria cases are deemed the main reason for the high number of recorded cases in the country^5,6^, but little is known of the contribution of sub-microscopic infection to the current status of malaria in the country. Sub-microscopic infections refer to infections that are too small to be detected by an ordinary light microscope^7^. Sub-microscopic malaria infections of *Plasmodium falciparum* are common in many endemic populations, particularly in low-transmission areas aiming for elimination^8^. These infections are of particular relevance to elimination programs, as they can persist for a number of months without any symptoms that would prompt seeking treatment^7,8^. While light microscopy examination of blood slides is the main method of detecting malaria infection, it has limited sensitivity and more sensitive methods such as polymerase chain reaction (PCR) are needed to detect sub-microscopic infections^8^.

According to Raman et al.^9^, detection of sub-microscopic (sub-patent) infections in South Africa communities is one of the key challenges that need to be addressed for South Africa to drive closer and ultimately achieve the desired elimination status,especially since these have the potential for further infection transmissibility^10^. Hermsen et al.^11^ defined sub-microscopic infections as “low-density *Plasmodium* infections detected only by molecular methods” and it has been demonstrated that sub-microscopic cases are identified in greater number among adults in low-endemic settings, similar to what is found in South Africa^8,12^. Prevalence of sub-microscopic infections^13^ in low malaria transmission areas has also been identified as serious threat in malaria elimination programmes^14,15^. However, core to the issue of sub-microscopic case detection and malaria elimination is the diagnostic approach. Malaria diagnostic method in South Africa is largely built around use of RDT. The operational principle of RDT as a malaria diagnostic tool is based on the identification of the antigens lactate dehydrogenase (LDH), aldolase or histidine-rich proteins in the parasite. LDH and aldolase antigens have been identified in all species of malaria while histidine-rich protein is specific for *Plasmodium falciparum*^16^. Deletion of the *Pfhrp* gene can lead to false negative reporting of RDT tests, of which *Pfhrp2* and *Pfhrp3* gene deletion has been widely reported in South America but less in Africa and Asia^17^, Irene^18^.

In preparation for the elimination stage, Solomon Islands, made use of a molecular based diagnostic method for the identification of sub-microscopic cases in one of the provinces. It effectively assisted with identifying underlining prevalence based on sub-microscopic infections and gave direction on approaching the elimination target^19^. An appropriate focus of resources and the drive to identify and deal with sub-microscopic infections as well as false negative RDT arising from deleted *Pfhrp* genes become important in view of the elimination target which has been set for 2025. We propose that in low transmission settings, false negative rapid diagnostic tests may contribute to an increase in sub-microscopic malaria infections. In addition, we hypothesize that the presence of *Pfhrp2/3* gene deletions has a direct correlation with sub-microscopic malaria. This study therefore aims to conduct a molecular assessment of sub-microscopic infections and also determine the prevalence of deleted *Pfhrp2* and *Pfhrp3* genes in low transmission districts of Kwa Zulu Natal province in South Africa using RDT and conventional PCR.

## Results

### The demographic outcome of the participants

Out of 354 completed responses, 197 were female (55.6%) and 157 were male (44.4%). A total of 190 (53.7%) belonged to the age group 25–47 years old, thus constituting the majority of the study participants. Furthermore, 238 (67.3%) were from urban while 116 (32.7%) lived in rural areas. 229 (64.6%) of the study participants have inter-district travel history, 29 (8.3%) have inter-province travel history while 6 (1.8%) had cross-border travel within Africa and 1 (0.3%) travelled outside Africa (Supplementary Table 2).

### Analysis of sub-microscopic infections

Following the testing of all the RDT, Table 1 showed that 4.2% (15/354) came out as positive while 95.6% (339/354) tested negative. The majority of the negative RDT result was from Ethekwini district 206 (X^2^ = 15.7, P < 0.001), with Ugu district having 60 (X^2^ = 141.5, P < 0.001), Uthungulu 45 (X^2^ = 5.7182.9, P < 0.001) and Ilembe 28 (X^2^ = 236.3, P < 0.001). All the RDT samples from Umkhuyakude accounting for 4.2% (15/354) tested positive. 339 RDT negative samples were sent for molecular test in the laboratory to validate the presence or absence of *Pfhrp2*, *Pfhrp3* and their flanking genes.

**Table 1:** Outcome distribution of RDT, PCR and *msp-1*gene amplification.

Location	RDT negative n = 339	${X}^{2}$	*18S rRNA* amplified and confirmed by sequencing	${X}^{2}$	*msp1* amplified n = 19	${X}^{2}$
Ilembe	28 (8.3%)	236.3 (< 0.001)	7 (2.1%)	311.6 (< 0.001)	6 (31.6%)	2.6 (0.108)
Uthungulu	45 (13.3%)	182.9 (< 0.001)	6 (1.8%)	315.4 (< 0.001)	6 (31.6%)	2.6 (0.108)
Ugu	60 (17.7%)	141.5 (< 0.001)	5 (1.5%)	319.3 (< 0.001)	5 (26.3%)	4.3 (0.039)
Ethekwini	206 (60.8%)	15.7 (< 0.001)	2 (0.6%)	331.0 (< 0.001)	2 (10.5%)	11.8 (0.001)

### The outcome of molecular tests

The nested reaction was used to screen for the *18S rRNA* gene amplification among the RDT negative cassettes and it showed 9.4% (32/339) to be positive for *P. falciparum*. However, following a further analysis with Sanger sequencing, 5.9% (20/339) were noted to have a good chromatogram appearance. After subjecting these 20 samples to quality test by screening for *msp1*, 5.6% (19/339) of the samples were found suitable.

### Analysis of *pfhrp2* and flanking genes

A total of 94.7% (18/19) of the isolates tested negative for the *Pfhrp2* gene with amplification shown only demonstrated in one sample (5.3%). In total, 89.5% (17/19) of the isolates showed deletion of the flanking gene, MAL7P1.230; while 84.2% gene deletions was demonstrated in the flanking gene MAL7P1.228 (Table 2).

**Table 2:** *Pfhrp2/3* and the flanking genes deletion outcome.

Location	*Pfhrp2* Exon		MAL7P1.230		MAL7P1.228		*Pfhrp3* Exon		MAL13P1.475		MAL13P1.485	
	+	−	+	−	+	−	+	−	+	−	+	−
Ilembe	0	6	0	6	2	4	0	6	0	6	0	6
Uthungulu	0	6	1	5	1	5	0	6	0	6	0	6
Ugu	0	5	0	5	0	5	0	5	0	5	0	5
Ethekwini	1	1	1	1	0	2	0	2	1	1	1	1
Total	5.3% (1/19)	94.7% (18/19)	10.5% (2/19)	89.5% (17/19)	15.8% (3/19)	84.2% (16/19)	0% (0/19)	100% (19/19)	5.3% (1/19)	94.7% (18/19)	5.3% (1/19)	94.7% (18/19)

‘+’ means positive; ‘−’ means negative.

### Analysis of *pfhrp3* and flanking genes

All the samples that amplified for both *18S rRNA* and *msp-1* were tested for the *pfhrp3* amplification. None of the screened samples showed amplification for *pfhrp3* (100% gene deletion). There was 94.7% demonstration of gene deletion of each of the flanking genes MAL13P1.475 and MAL13P1.485 (Table 2).

### Phylogenetic analysis

Twenty out of the twenty seven amplicons that were sequenced came back with good chromatogram results. Blast analysis of the *18S rRNA* in this study showed 93–100% similarity with other *P. falciparum 18S rRNA* in the NCBI database. A Maximum likelihood (ML) phylogenetic tree (Supplementary Fig. 5) was created using the sequences of the twenty *P. falciparum* that were generated in this study together with the ones that were retrieved from NCBI for *P. falciparum* isolates from India, China, United Kingdom, Myanmar, Mexico, Nigeria and Iran. Based on the phylogenetic tree, *P. falciparum* sequences generated from this study clustered together though in the same clade with other *P. falciparum* from China, India, United Kingdom (UK), Iran, and Myanmar.

## Discussion

Malaria RDT serves as a major point-of-care (POC) diagnostic tool in South Africa, and it is widely used in many parts of the country due to ease of application. However, limitations such as false negativity is associated with RDT use, hence, the use of microscopy as an adjunct tool in confirmation of a positive diagnosis of malaria. Although reliable, the accuracy of microscopy is dependent on a lot of factors which include availability of trained individuals, quality and the sensitivity of microscope used as well as the sample handling technique. In this study which was conducted in a malaria low transmission setting, we identified RDT positivity of 4.2% (15/354). Previous study in the province reported a prevalence rate of 2% for local cases^6^. Our finding aligns with the trend reported in low transmission settings where malaria positivity decreases as the elimination stage sets in Laban et al.^20^. By inference, this is a possible reflection of the effectiveness of the different methods that have been applied during the malaria elimination campaigns in the province. It could also be a reflection of regular improvement in the quality control methods.

No doubt, microscopy detection of *plasmodium* is a widely accepted “gold standard” for malaria diagnosis, but it is unable to detect low levels of the parasite, known as sub-microscopic infections^21^. However, more advanced molecular techniques such as polymerase chain reaction (PCR) are capable of detecting these infections with greater sensitivity, making them more suitable for routine diagnosis^21,22^. Another study suggested that nucleic acid methods are typically the most effective diagnostic tools for identifying Plasmodium species^23^.

Considering the low transmission setting of the study, we chose to use a more sensitive test as prescribed in the recommendation-3 of the World Health Organization Evidence Review Group on Malaria diagnosis in Low Transmission Settings^24^, by confirming the presence of the *P. falciparum* using the conventional PCR followed by Sanger sequencing of the positive amplicons. Following the analysis of the sub-microscopic infections, 9.4% of the negative RDT samples tested positive with conventional PCR, thus confirming the inherent shortfalls with the use of RDT as surveillance diagnostic tool in settings that are nearing elimination^25,26^. This justifies the call by the World Health Organization for the use of molecular tool during surveillance in pre- and post-elimination settings^27^. In fact, a study conducted in low transmission setting of Swaziland (eSwatini) by Ranadiwe et al.^26^ affirmed the low sensitivity of RDT. Thus, as a country that looks forward to eliminating malaria, a practical step in a positive direction requires deploying a more efficient tool that can detect sexual and asexual stages of the parasites, particularly for surveillance in very low transmission areas. Molecular tools that operate on the principle of nucleic acid detection have been demonstrated to be efficient in this regard; an example of this is Loop-mediated isothermal amplification (LAMP). LAMP operates on the principle of gene amplification, but it is less complex when compared with conventional or real time PCR^24,28^. This relative advantage makes it a tool for consideration in large scale surveillance that is focused on identification of sub-microscopic cases,and this can be combined effectively with focal mass drug administrative program.

Although South Africa is designated as low transmission, imported cases from neighbouring countries such as Mozambique and Zimbabwe^29^ are still common. Some of our study participants also had a travel history outside Africa. Analysis of migration (75%) among the study participants (Supplementary Table 2) indicates inter-district, inter-province and international movement accounted for 64.6%, 8.3% and 2.1% respectively. Studies have shown that migration has the potential for spreading malaria^30–32^. There is the possibility that the identified sub-microscopic cases were actually spreading from rural to urban and vice-versa. In that regard, there is need to put in place measures that will curtail this spread among locations that have been certified as malaria-free and thus calling for priority into investing in research and strengthening of already existing elimination approach.

Based on the result obtained in the positive samples from the sub-microscopic cases, we built a Maximum-Likelihood phylogenetic tree to determine how genetically related and diverse are the isolates; and found close evolutionary and possible common ancestry between one of our *P. falciparum* isolates (OP341871.1) and those of Asian origin. Despite this close relatedness, the diversity in the local population was obvious. Genetic diversity has been determined in *P. falciparum* using different markers including the *merozoite surface protein (msp)* genes^33^ and related to transmission in different environments. Abdelraheem et al.^34^ reported a high genetic diversity among *Plasmodium* that were imported from different countries and implicated India as major importer of *Plasmodium viva.* According to Xu et al.^35^ there was high density in *P. falciparum* isolates among returning Chinese migrant workers from Africa, thus supporting the claim of parasite exchange between the two continents of Africa and Asia. Although China has recently received a WHO certification of malaria elimination, there is a claim that imported malaria cases are often by Chinese workers in South Africa (and other parts of Africa) and these serve as vessels for the importation of the malaria parasite^36,37^. These might explain the close genetic relatedness in our *P. falciparum* isolates and those from China and India.

Since the initial identification of *Phrp2/3* gene deletion in Peru^38^ and the subsequent reports by many countries^39,40^, the growing concerns call for a widespread study in both high and low-transmission settings. In general, most documented reports are mainly studies that were carried out in regions that are malaria endemic as obtained in sub-Saharan Africa, Asia and South America^16,41^. Our study identified non-amplification of a large proportion of the *Pfhrp2* and the *Pfhrp3* genes − 94.7% and 100% respectively. This finding is in tandem with the outcome of previous studies that were carried out in places with a low prevalence of malaria (less than 1 per 1000 population) like Honduras (*Pfhrp2*—0%, *Pfhrp3*—96.2%) and Guatemala (*Pfhrp2*—14.3%, *Pfhrp3*—90.5%) which recorded high gene deletions, especially the *Pfhrp3* gene^38,39^. Also, a meta-analysed mean prevalence by Nyataya et al.^16^ showed that there is a higher mean incidence of *Pfhrp3* in comparison with *Pfhrp2*. The documented African studies were mainly from the malaria-endemic countries in the sub-Saharan region and no study has been documented so far from the countries where the malaria endemic level is low^41^. To the best knowledge of the authors, this is the first study in a low endemic region located in sub-Saharan Africa. According to Berzosa et al.^42^, there is a possibility of the selection of *Pfhrp2* gene deletion with the over-use of Hrp-based RDTs. The identification of a large proportion of the *Pfhrp 2/3* gene deletion in places which have been regarded as almost free of malaria transmission makes a pointer to the need for a closer look into the surveillance diagnostic methods that are currently being applied.

Low malaria parasite level is another reason associated with false-negative RDT, especially in low endemicity settings^43^. To further shed light on this, study by Beshir et al.^44^ in which the relationship between the parasitemia level and the gene deletion was modelled, it was noted that “the probability of RDT positivity for *Pfhrp2* negative samples in the presence of *Pfhrp3* samples increased with parasitaemia and was close to the one for *Pfhrp2* positive samples in the presence of *Pfhrp3* positive samples for parasitaemia levels > 1000 μl. Such proximity of the two probabilities together with the reduced number of *Pfhrp2* negative samples in the presence of *Pfhrp3* amplified samples implied that they were not statistically different as a function of parasitaemia”. Bharti et al.^45^ further reported that 48% of subjects with high parasite density tested negative with RDT, thus indicating that an exclusively low parasitemia is not enough to explain false negative RDT. This is also in line with the report by Wurtz et al.^46^ who identified no difference between the group with deletion and those without deletion. Although parasitemia level was not evaluated in this study it may not have a significant impact on the noted outcomes since good concentration (1.1–2.9 ng/µl) as well as quality DNA was ensured in the positive samples that were used for the final amplification step.

Part of the study limitation identified include the fact that even though the authors used the World Health Organization recommendation of molecular based diagnostic method in the low transmission setting, the use of microscopy could still be considered in future studies. However, the number of trained personnel in malaria microscopy has fallen drastically in the past few years in South Africa thus posing challenges to microscopy-based diagnosis. Furthermore, since this study was done using five KwaZulu Natal districts, it will be worthwhile if a national survey be conducted to give the overall picture and prevalence rate of both sub-microscopic cases and deleted *Pfhrp2/3* gene. This will assist with national malaria program planning as well as ways of recommendations as per World Health Organization policy.

In conclusion, the study identified the presence of sub-microscopic *P. falciparum* infections *Pfhrp2/3* gene deletions in KwaZulu Natal, South Africa where malaria is in low transmission. The positivity rates obtained in both instances were in consonance with that found in low transmission zones and are significant at a time when the malaria elimination agenda is looking forward to 2025. A high rate of migration also characterized the study with the phylogenetic analysis suggesting a possible evolutionary connection between the strain from South Africa and that of China United Kingdom, Iran, Myanmar and India. Having invested so much in machinery and manpower to reach the pre-elimination status, our study findings are indications that South Africa needs to consider a national study on the prevalence of deleted *Pfhrp2/3* genes. In addition, bearing in mind the possibility of the establishment of cycles of infection that could result from sub-microscopic residuals in the presence of appropriate conditions, intensifying efforts to achieve elimination must not undermine consideration of an application of molecular-based diagnostics which have greater potential to overcome the limitations of RDT and microscopy and also provide more usefulness in the disease surveillance.

## Method

### Study area

**Figure 1:**
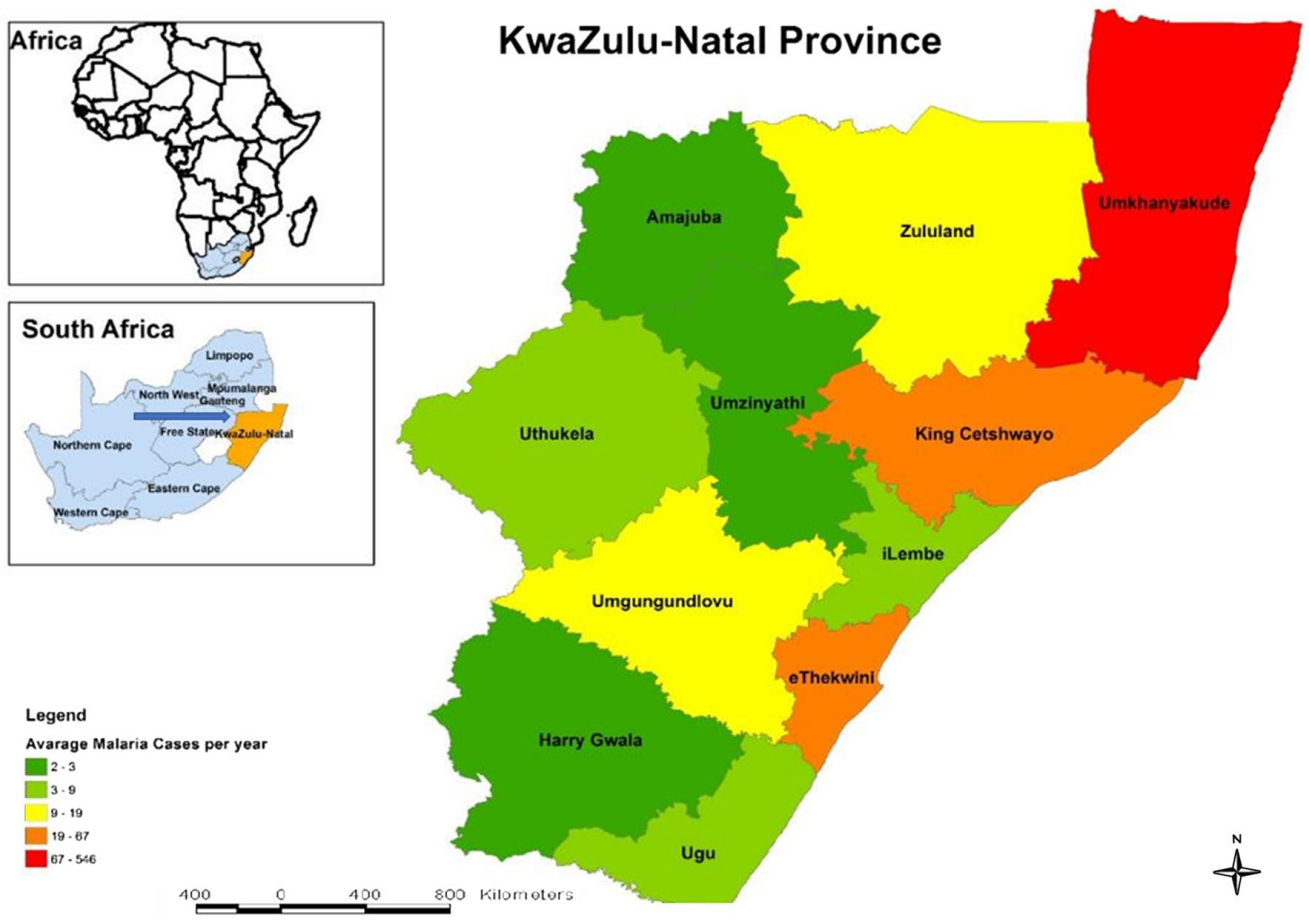
Map showing endemic provinces of South Africa and KwaZulu Natal district.

The study was conducted in KwaZulu Natal which is known for malaria transmission where *Plasmodium falciparum* is the predominant species (Fig. 1). It is located on the coordinates 28.5306°S, 30.8958°E with warm and subtropics climates and a land mass of 94,000km^2^ with eleven districts municipalities which are mostly rural, and harbour a population of about 11.1 million. The province is among the three in the country that still records very-low to a low incidence of malaria cases.

### Study population

Samples were collected in the following districts of KwaZulu Natal: Ethekwini, Ilembe, Ugu, Uthungulu, and Umkhumyakude (Fig. 1). The health facilities are situated in rural areas, with the exception of the Ethekwini district, where they are situated in urban areas. The study received ethical approval from the Biomedical Research Ethics committee of the University of KwaZulu Natal South Africa (BREC/00001815/2020). Participants with symptoms suggestive of malaria were recruited from health facilities in the listed districts after obtaining informed consents from interested individuals. Accompanied minors are allowed to participate following parental or guardians’ informed consents. Individuals with history suggestive of bleeding disorder were excluded. All methods were carried out following relevant guidelines and regulations.

### Sample size determination

Since there were more than 10,000 participants in the study, the sample size was determined using Fisher's formula^47^. Assuming a 95% confidence interval is equal to 1.96, a 5% acceptable margin of error, and a maximum variability of 50% given the unknown prevalence, one can estimate the prevalence of the desired outcomes. 384 patients will be needed as a sample size based on the statistical parameters combined.

Each medical facility's patients were purposefully chosen until the calculated sample size was reached for each facility. The power of the sample (1 − β) and the (%) chance of detecting differences in the study were set at 80%. However, out of 384 participants approached, only 354 accepted to participate making it a 92.2% response rate bearing in mind that “a response rate of ≥ 80% was expected”^48^.

Furthermore, the study used both purposive and convenient sampling to select people who agreed to participate in the survey. According to Teddlie and Yu^49^ and Radhakrishnan^50^. Convenience sampling involves selecting samples that are both readily available and willing to participate in a study, whereas purposive sampling is predicated on the idea that researcher knowledge of the population can be used to select sample members. The sampling period for our study was from March 2021 to January 2022 which was the period that witnessed a high incidence of COVID-19 in South Africa and to some extent limiting the accessibility to different health facilities.

### RDT kit testing and data collection

RDT kit (ICT MALARIA DUAL Test—Malaria Pf/PAN antigen RDT, Cape Town, South Africa) was used for the testing. It was reported by the manufacturer to have been compared with microscopy based on 200 parasites/ml and an International Laboratory evaluation against PCR showing a sensitivity of 96.8% and 96.3% for non *P. falciparum* and *P. falciparum* respectively and specificity of 99.7%. Following approved consent, the participant’s finger was cleaned with an alcohol-based solution and allowed to air dry. A sterile lancet was used for the finger prick and 5 µl of blood was collected in the blood collection device supplied by the manufacturer and transferred to the cassette following which five drops of reaction buffer were placed in the large base well of the cassette and the outcome read in 15–30 min. The test outcomes were recorded as positive, negative or invalid and the used RDT kits were stored on-site at room temperature in dry pouches containing desiccants. This was followed by the administration of a short questionnaire containing the demographic data of the participants. The questionnaires were taken for statistical analysis while the RDT cassettes from these study sites were taken for further PCR testing in the laboratory.

### DNA extraction and amplification

Each RDT cassette was opened and the nitrocellulose strip was carefully removed under sterile condition as recommended by an established protocol^51^. The proximal segment of the nitrocellulose strip was dissected and used for DNA extraction. The process of DNA extraction was done using the Zymo kit (*Quick*-DNA™ Miniprep-Plus Kit) based on the manufacturer’s guidelines for nucleated blood samples with an elution volume of 50 µL. The purity and concentrations of DNA were assessed using Nanodrop 2000. Samples were stored at minus 20 degrees centigrade until use. Nitrocellulose strips devoid of DNA were used for the control test.

Thermo Electron® PX2 (HBPX2) thermal cycler was used for the primary and nested PCR procedure which was performed on the DNA extracts using published pairs of primers by Somé et al.^52^—(rPLUf 5′-TTAAAATTGTTGCAGTTAAAACG-3’, rPLUr 5′-CCTGTTGTTGCCTTAAACTTC-3′; nested PCR rFALf 5′-TTAAACTGGTTTGGGAAAACCAAATATATT-3′, rFALr 5′-ACACAATGAACTCAATCATGACTACCCGTC-3′). Each reaction ran in total volume of 25 μl volume with 1 μl of forward and reverse primer in each case and 12.5 μl master mix added (1 × AmpliTaq Gold buffer, 1.5 mM MgCl 2, 0.25 mM dNTPs, 1 U of AmpliTaq Polymerase) to each reaction. Nuclease free water was 9.5 μl and 8.5 μl in the primary and the nested reactions respectively. 2 μl of template DNA was added in primary while 1 μl of the PCR product of the first PCR was used as the template for the second PCR. The primary cycle of 35 cycles comprised an initial denaturation at 95 °C for 3 min, followed by final denaturation at 95 °C for 30 s, 53.1 °C 60 s and 72 °C for 60 s, and final elongation at 72 °C for 5 min. The nested PCR ran in 30 cycles and began with denaturation step at 95 °C for 5 min and extension at 95 °C for 30 s, 58.7 °C for 60 s, 72 °C for 60 s. The final elongation was at 72 °C for five minutes. The resultant amplicons from the secondary PCR were analysed on 1.5 (w/v) agarose gel electrophoresis at 70 V for 40 min and visualised under ultraviolet light using a Bio-RadChemiDoc™ MP System (Bio-Rad, US) with a baseline expected band of230bp. This was subsequently followed by Sanger sequencing of the PCR products with both forward and reverse primers at the Central Analytical Facility of the University of Stellenbosch, South Africa.

### PCR confirmation of *P. falciparum* infection

In order to ensure that samples that will be used in the amplification of *Pfhrp2*, *Pfhrp3* and their neighbouring genes have an adequate quality of genomic DNA; PCR amplification of the k1-allele of *P. falciparum* merozoite surface protein-1 (*pfmsp1*) was also performed on samples that tested positive for *18S rRNA* gene and confrimed by Sanger’s sequence. The processes of these gene amplifications were done using the method that was earlier described by Somé et al.^52^. The primers and annealing temperature used for both reactions are stated in Supplementary table 1. The primary cycle of 29 cycles comprised an initial denaturation at 94 °C for 30 s, 94 °C for 30 s, 53.1 °C for 60 s (annealing) and elongation at 68 °C for 60 s, and final elongation at 72 °C for 5 min. The nested PCR ran in 32 cycles and began with the denaturation step at 94 °C for 30 s, followed by 94 °C for 30 s, 68 °C for 5 min, 68 °C for 60 s, and final elongation was at 72 °C for five minutes.

For the *Pfmsp1-k1*, each of the primary and nested sample reaction was in a total volume of 25 μl comprising 2 μl of forward primer, 2 μl of reverse primer, 6.5 μl of nuclease free water and 12.5 μl Dreamtaq Green PCR Master mix (DreamTaq DNA polymerase, 2X Dreamtaq Green buffer, dATP, dCTP, dGTP and dTTP, 0.4 mM each, and 4 mM MgCl_2_, Thermo Fisher Scientific), 2 μl of genomic DNA was added in each reaction. The resultant PCR products were analysed on 1.5 (w/v) agarose gel electrophoresis at 70 V for 40 min and visualised under ultraviolet light using a Bio-RadChemiDoc™ MP System (Bio-Rad, US).

### PCR detection of *pfhrp2*, *pfhrp3* and their flanking genes

Samples that were RDT negative but amplified for both *18S r RNA* gene and *Pfmsp1*were selected for detection of *Pfhrp2* and *Pfhrp3* deletion. The nested PCR protocol described by Abdallah et al.^39^ was used for the amplification of exon1, the intron, and exon 2 of *Pfhrp2* and *Pfhrp3*. Furthermore, the same protocol was used to assess the amplification of MAL7P1.230 and MAL7P1.228. The primers and annealing temperature used have been listed in Supplementary file, Table 1. Each reaction was performed in a total volume of 25 μl which consisted of forward primer (2μ), reverse primer (2μ), nuclease-free water (6.5 μl) and 12.5 μl Dreamtaq Green PCR Master mix (DreamTaq DNA polymerase, 2X Dreamtaq Green buffer, dATP, dCTP, dGTP and dTTP, 0.4 mM each, and 4 mM MgCl_2_, Thermo Fisher Scientific). The *P. falciparum* parasite strain 3D7 was used as a positive control for the PCR analyses of *Pfhrp2*, *Pfhrp3* as well as their flanking genes.

### Sequence analysis and phylogeny

The forward and reverse sequences were edited using the BioEdit version 7.2 and these were submitted to the National Centre for Biotechnology Information (NCBI) and an accession number issued (OP341852–OP341871). Sequence similarity through Blastn analysis was performed and orthologous sequences were retrieved from the Gene Bank. Multiple sequence alignment of our sequences and those retrieved from the GenBank was carried out with Cluster-W program in MEGA version. Also, the best DNA model of the aligned sequences was evaluated using the AIC scores and phylogenetic tree based on Maximum Likelihood (ML) method was constructed with MEGA version 6.0 using Hasegawa-Kishino-Yano (HYK) model^53^ with bootstrap of 1000. *Babesia bigemina* was used as an out-group to root the phylogenetic tree.

### Statistical analysis

Statistical analysis was done to determine positivity outcome of tested RDT and PCR. Relationships between variables were assessed by Chi-square test or Fisher’s exact test for categorical variables. The level of significance was set at $\alpha =0.01$. The prevalence of the deleted *Pfhrp2*, *Pfhrp3*, and flanking gene was calculated by dividing the number of isolate in which there are deleted genes by the total number of isolates that were amplified by both *18S rRNA* and *msp-1*. Statistical analyses were performed using the software package SPSSv.21.0. and Stata 14 (Stata Corp., College Station, TX, USA).

### Ethical approval and consent to participate

The ethic for this study was approved by the Biomedical Research Ethics committee of the University of KwaZulu Natal South Africa with ethic approval number BREC/00001815/2020.

### Supplementary Information

Supplementary Information.

## Data Availability

The genomic sequencing data generated and/or analyzed during this current study are available through the National Centre for Biotechnology Information (NCBI)—accession numbers OP341852–OP341871. Upon reasonable request, the datasets used in this study are available from the corresponding author.

## References

[1] Talapko J, Škrlec I, Alebić T, Jukić M, Včev A (2019). Malaria: The past and the present. Microorganisms.

[2] Zhang YL, Pan WQ (2022). The world's first malaria vaccine: Hope and challenge. Chin. J. Schistosom. Control.

[3] Maharaj R, Morris N, Seocharan I (2012). The feasibility of malaria elimination in South Africa. Malar. J..

[4] South Africa National Department of Health. *Malaria Elimination Strategic Plan for South Africa 2019–2023*, 1–84 (2019).

[5] Moonasar DM, Davies C, Balawanth R, Misiani E, Shandukani MB, Raman J, Pillay YG (2021). Progress, challenges and priorities for malaria elimination in South Africa. Trans. R. Soc. S. Afr..

[6] Raman J, Gast L, Balawanth R, Tessema S, Brooke B, Maharaj R, Moonasar D (2020). High levels of imported asymptomatic malaria but limited local transmission in KwaZulu-Natal, a South African malaria-endemic province nearing malaria elimination. Malar. J..

[7] Vareta, J. *et al*. Submicroscopic malaria infection is not associated with fever in cross-sectional studies in Malawi. *Malar. J.* **19**, 1–8 (2020). 10.1186/s12936-020-03296-4 PMC7322713 32600362

[8] Okell L, Bousema T, Griffin J (2012). Factors determining the occurrence of submicroscopic malaria infections and their relevance for control. Nat. Commun..

[9] Raman J, Morris N, Frean J (2016). Reviewing South Africa’s malaria elimination strategy (2012–2018): Progress, challenges and priorities. Malar. J..

[10] Golassa L, Enweji N, Erko B, Aseffa A, Swedberg G (2013). Detection of a substantial number of sub-microscopic *Plasmodium falciparum* infections by polymerase chain reaction: a potential threat to malaria control and diagnosis in Ethiopia. Malar. J..

[11] Hermsen CC, Telgt DS, Linders EH, van de Locht LA, Eling WM, Mensink EJ, Sauerwein RW (2001). Detection of *Plasmodium falciparum* malaria parasites in vivo by real-time quantitative PCR. Mol. Biochem. Parasitol..

[12] Whittaker C, Slater H, Nash R, Bousema T, Drakeley C, Ghani AC, Okell LC (2021). Global patterns of submicroscopic *Plasmodium falciparum* malaria infection: Insights from a systematic review and meta-analysis of population surveys. Lancet Microb..

[13] Fradejas I, Rubio JM, Martín-Díaz A (2019). Prevalence of submicroscopic malaria infection in immigrants living in Spain. Malar. J..

[14] Kaura T, Kaur J, Sharma A, Dhiman A, Pangotra M, Upadhyay AK, Grover GS, Sharma SK (2019). Prevalence of sub microscopic malaria in low transmission state of Punjab: A potential threat to malaria elimination. J. Vector Dis..

[15] Lin JT, Saunders DL, Meshnick SR (2014). The role of submicroscopic parasitemia in malaria transmission: What is the evidence?. Trends Parasitol..

[16] Nyataya J, Waitumbi J, Mobegi VA, Noreddin A, El Zowalaty ME (2020). Plasmodium falciparum histidine-rich protein 2 and 3 gene deletions and their implications in malaria control. Diseases.

[17] Murillo Solano C, Akinyi Okoth S, Abdallah JF, Pava Z, Dorado E, Incardona S, Barnwell JW (2015). Deletion of *Plasmodium falciparum* histidine-rich protein 2 (pfhrp2) and histidine-rich protein 3 (pfhrp3) genes in Colombian parasites. PLoS ONE.

[18] Molina-de la Fuente I, Pastor A, Herrador Z, Benito A, Berzosa P (2021). Impact of *Plasmodium falciparum* pfhrp2 and pfhrp3 gene deletions on malaria control worldwide: A systematic review and meta-analysis. Malar. J..

[19] Harris I, Sharrock WW, Bain LM (2010). A large proportion of asymptomatic *Plasmodium* infections with low and sub-microscopic parasite densities in the low transmission setting of Temotu Province, Solomon Islands: challenges for malaria diagnostics in an elimination setting. Malar. J..

[20] Laban NM, Kobayashi T, Hamapumbu H, Sullivan D, Mharakurwa S, Thuma PE, Shiff CJ, Moss WJ, Southern Africa International Centers of Excellence for Malaria Research (2015). Comparison of a PfHRP2-based rapid diagnostic test and PCR for malaria in a low prevalence setting in rural southern Zambia: Implications for elimination. Malar. J..

[21] Rahi M, Sharma R, Saroha P, Chaturvedi R, Bharti PK, Sharma A (2022). Polymerase chain reaction-based malaria diagnosis can be increasingly adopted during current phase of malaria elimination in India. Am. J. Trop. Med. Hyg..

[22] Abad P, Marín-García P, Heras M, Fobil JN, Hutchful AG, Diez A, Puyet A, Reyes-Palomares A, Azcárate IG, Bautista JM (2022). Microscopic and submicroscopic infection by *Plasmodium falciparum*: Immunoglobulin M and A profiles as markers of intensity and exposure. Front. Cell Infect. Microbiol..

[23] Tedla M (2019). A focus on improving molecular diagnostic approaches to malaria control and elimination in low transmission settings: Review. Parasit. Epidemiol. Control..

[24] World Health Organization. *Malaria Policy Advisory Committee Meeting 12–14 March 2014, WHO HQ, Geneva Session 10*. (2014). https://www.who.int/publications/m/item/malaria-policy-advisory-committee-march-2014. Accessed Feb 2024.

[25] McMorrow ML, Aidoo M, Kachur SP (2011). Malaria rapid diagnostic tests in elimination settings: Can they find the last parasite. Clin. Microbiol. Infect..

[26] Ranadive N, Kunene S, Darteh S, Ntshalintshali N, Nhlabathi N, Dlamini N, Hsiang MS (2017). Limitations of rapid diagnostic testing in patients with suspected malaria: A diagnostic accuracy evaluation from Swaziland, a low-endemicity country aiming for malaria elimination. Clin. Infect. Dis..

[27] World Health Organization. *Disease Surveillance for Malaria Elimination: An Operational Manual*. (2012). https://www.who.int/iris/bitstream/handle/10665/44852/9789241503334_eng.pdf;jsessionid=FCC0726D9D8F9DCF2C1FB638D86CAE7F?sequence=1. Accessed Oct 2022.

[28] Notomi T, Mori Y, Tomita N (2015). Loop-mediated isothermal amplification (LAMP): Principle, features, and future prospects. J. Microbiol..

[29] Njau J, Silal SP, Kollipara A (2021). Investment case for malaria elimination in South Africa: A financing model for resource mobilization to accelerate regional malaria elimination. Malar. J..

[30] Martens P, Hall L (2000). Malaria on the move: Human population movement and malaria transmission. Emerg. Infect. Dis..

[31] Pousibet-Puerto J, Lozano-Serrano AB, Soriano-Pérez MJ (2021). Migration-associated malaria from Africa in southern Spain. Parasit. Vectors.

[32] Rodrigues PT, Valdivia HO, de Oliveira TC (2018). Human migration and the spread of malaria parasites to the New World. Sci. Rep..

[33] Olasehinde GI, Yah CS, Singh R, Ojuronbge OO, Ajayi AA, Valecha N, Abolaji AO, Adeyeba AO (2012). Genetic diversity of *Plasmodium falciparum* field isolates from south western Nigeria. Afr. Health Sci..

[34] Abdelraheem MH, Bansal D, Idris MA (2018). Genetic diversity and transmissibility of imported *Plasmodium vivax* in Qatar and three countries of origin. Sci. Rep..

[35] Xu C, Huang B, Wei Q, Li J, Kong X, Xiao T, Sun H, Zhao G, Yan G, Gong M, Yin K (2022). High genetic diversity in *Plasmodium falciparum* isolates among Chinese migrant workers returnee from Africa. Parasitol. Res..

[36] Lai S, Sun J, Ruktanonchai NW, Zhou SYJ, Routledge I, Wang L, Zheng Y, Tatem AJ, Li Z (2019). Changing epidemiology and challenges of malaria in China towards elimination. Malar. J..

[37] Wang D, Lv S, Ding W, Lu S, Zhang H, Kassegne K, Xia S, Duan L, Ma X, Huang L, Gosling R, Levens J, Abdulla S, Mudenda M, Okpeku M, Matengu KK, Diagbouga PS, Xiao N, Zhou X-N (2022). Could China’s journey of malaria elimination extend to Africa?. Infect. Dis. Poverty.

[38] Gamboa D, Ho MF, Bendezu J, Torres K, Chiodini PL, Barnwell JW, Incardona S, Perkins M, Bell D, McCarthy J (2010). A large proportion of *P. falciparum* isolates in the Amazon region of Peru lack pfhrp2 and pfhrp3: Implications for malaria rapid diagnostic tests. PLoS ONE.

[39] Abdallah JF, Okoth SA, Fontecha GA (2015). Prevalence of pfhrp2 and pfhrp3 gene deletions in Puerto Lempira, Honduras. Malar. J..

[40] Gupta H, Matambisso G, Galatas B, Cistero P, Nhamussua L, Simone W (2017). Molecular surveillance of pfhrp2 and pfhrp3 deletions in *Plasmodium falciparum* isolates from Mozambique. Malar. J..

[41] Agaba BB, Yeka A, Nsobya S (2019). Systematic review of the status of *pfhrp2* and *pfhrp3* gene deletion, approaches and methods used for its estimation and reporting in *Plasmodium falciparum* populations in Africa: review of published studies 2010–2019. Malar. J..

[42] Berzosa P, de Lucio A, Romay-Barja M (2018). Comparison of three diagnostic methods (microscopy, RDT, and PCR) for the detection of malaria parasites in representative samples from Equatorial Guinea. Malar. J..

[43] Koita OA, Doumbo OK, Ouattara A, Tall LK, Konaré A, Diakité M, Krogstad DJ (2012). False-negative rapid diagnostic tests for malaria and deletion of the histidine-rich repeat region of the hrp2 gene. Am. J. Trop. Med. Hyg..

[44] Beshir KB, Sepúlveda N, Bharmal J, Robinson A, Mwanguzi J, Busula AO, de Boer JG, Sutherland C, Cunningham J, Hopkins H (2017). *Plasmodium falciparum* parasites with histidine-rich protein 2 (pfhrp2) and pfhrp3 gene deletions in two endemic regions of Kenya. Sci. Rep..

[45] Bharti PK, Chandel HS, Ahmad A, Krishna S, Udhayakumar V, Singh N (2016). Prevalence of pfhrp2 and/or pfhrp3 gene deletion in *Plasmodium falciparum* population in eight highly endemic states in India. PLoS ONE.

[46] Wurtz N, Fall B, Bui K, Pascual A, Fall M, Camara C, Pradines B (2013). Pfhrp2 and pfhrp3 polymorphisms in *Plasmodium falciparum* isolates from Dakar, Senegal: Impact on rapid malaria diagnostic tests. Malar. J..

[47] Daniel WW (1999). Biostatistics: A Foundation for Analysis in the Health Sciences.

[48] Fincham JE (2008). Response rates and responsiveness for surveys, standards, and the Journal. Am. J. Pharm. Educ..

[49] Teddlie C, Yu F (2007). Mixed methods sampling a typology with examples. J. Mixed Methods Res..

[50] Radhakrishna G (2014). Sampling in mixed methods research. Int. J. Adv. Nur. Manag..

[51] Molecular Module. *Preparation of Rapid Diagnostic Tests (RDTs) for DNA extraction v1.1. WWARN Procedure*. (2011). https://www.wwarn.org/sites/default/files/attachments/procedures/MOL06_RDTsForDNAExtraction.pdf.

[52] Somé AF, Bazié T, Zongo I, Yerbanga RS, Nikiéma F, Neya C, Ouédraogo JB (2018). Plasmodium falciparum *msp1* and *msp2* genetic diversity and allele frequencies in parasites isolated from symptomatic malaria patients in Bobo-Dioulasso, Burkina Faso. Parasit. Vectors.

[53] Hasegawa M, Kishino H, Yano T (1985). Dating of the human-ape splitting by a molecular clock of mitochondrial DNA. J. Mol. Evol..

