# Supplementary material for: Molecular detection of sub-microscopic infections and Plasmodium falciparum histidine-rich protein-2 and 3 gene deletions in pre-elimination settings of South Africa
Source: Sci Rep. 2024 Jul 11;14:16024. doi: 10.1038/s41598-024-60007-8 (PMC11239831; doi:10.1038/s41598-024-60007-8)
Supplement: Supplementary file 1 — Supplementary Information. [file 41598_2024_60007_MOESM1_ESM.docx]

**SUPPLEMENTARY**

**Supplementary Table 1 Primer names and PCR details used for identification of deleted genes**

| Gene | Reaction | Primer  name | Sequence | Annealing  temperature | | Reference |
| --- | --- | --- | --- | --- | --- | --- |
| PF3D7_0831900 (MAL7P1.230) | Primary | 230 F1 | 5′ GAT ATC ATT AGA AAA CAA GAG CTT AG 3’ | 63 | Abdallah et al., 2015 | |
|  |  | 230 R | 5′ TAT CCA ATC CTT CCT TTG CAA CAC C 3′ |  | Abdallah et al., 2015 | |
|  | Nested | 230 F | 5′ TAT GAA CGC AAT TTA AGT GAG GCA G 3′ | 65 | Abdallah et al., 2015 | |
|  |  | 230R | 5′ TAT CCA ATC CTT CCT TTG CAA CAC C 3′ |  | Abdallah et al., 2015 | |
| PF3D7_0831700 (MAL7P1.228) | Primary | 228 F | 5′ AGA CAA GCT ACC AAA GAT GCA GGT G 3′ | 60 | Abdallah et al., 2015 | |
|  |  | 228 R | 5′ TAA ATG TGT ATC TCC TGA GGT AGC 3′ |  | Abdallah et al., 2015 | |
|  | Nested | 228 F1 | 5′ CCA TTG CTG GTT TAA ATG TTT TAA G 3′ | 63 | Abdallah et al., 2015 | |
|  |  | 228R | 5′ TAA ATG TGT ATC TCC TGA GGT AGC 3′ |  | Abdallah et al., 2015 | |
| PF3D7_1372100, (MAL13P1.485) | Primary | 485 F | 5′ TTG AGT GCA ATGATG AGT GGA G 3′ | 60 | Abdallah et al., 2015  Abdallah et al., 2015 | |
|  |  | 485R | 5′ AAA TCA TTT CCT TTT ACA CTA GTG C 3′ |  | Abdallah et al., 2015 | |
|  | Nested | 485 F1 | 5′ GTT ACT ACA TTA GTG ATG CAT TC 3′ | 59 | Abdallah et al., 2015 | |
|  |  | 485 R | 5′ AAA TCA TTT CCT TTT ACA CTA GTG C 3′ |  | Abdallah et al., 2015 | |
| PF3D7_1372400 (MAL13P1.475) | Primary | 475 F | 5′ TTC ATG AGT AGA TGT CCT AGG AG 3′ | 55 | Abdallah et al., 2015 | |
|  |  | 475 R | 5′ TCG TAC AAT TCA TCA TAC TCA CC 3′ |  | Abdallah et al., 2015 | |
|  | Nested | 475 F | 5′ TTC ATG AGT AGA TGT CCT AGG AG 3′ | 61 | Abdallah et al., 2015 | |
|  |  | 475 R1 | 5′ GGA TGT TTC GAC ATT TTC GTC G 3′ |  | Abdallah et al., 2015 | |
| PfHRP2 Exon 1–2, PF3D7_0831800 | Primary | 2E12F1 | 5′ GGT TTC CTT CTC AAA AAA TAA AG 3′ | 55 | Abdallah et al., 2015 | |
|  |  | 2E12R1 | 5′ TCT ACA TGT GCT TGA GTT TCG 3′ |  | Abdallah et al., 2015 | |
|  | Nested | 2E12F | 5′ GTA TTA TCC GCT GCC GTT TTT GCC 3′ | 62 | Abdallah et al., 2015 | |
|  |  | 2E12R | 5′ CTA CAC AAG TTA TTA TTA AAT GCG GAA 3 |  | Abdallah et al., 2015 | |
| PfHRP3 Exon 1–2, PF3D7_1372200 | Primary | 3E12F1 | 5′ GGT TTC CTT CTC AAA AAA TAA AA 3′ | 53 | Abdallah et al., 2015 | |
|  |  | 3E12R1 | 5′ CCT GCA TGT GCT TGA CTT TA 3′ |  | Abdallah et al., 2015 | |
|  | Nested | 3E12F | 5′ ATA TTA TCG CTG CCG TTT TTG CT 3′ | 62 | Abdallah et al., 2015 | |
|  |  | 3E12R | 5′ CTA AAC AAG TTA TTG TTA AAT TCG GAG 3′ |  | Abdallah et al., 2015 | |
| MSP1 | Primary | M1-OF | 5’ CTAGAAGCTTTAGAAGATGCAGTATTG 3’ | 51 | Abdallah et al., 2015 | |
|  |  | M1-OR | 5’ CTTAAATAGTATTCTAATTCAAGTGGATCA 3’ |  | Abdallah et al., 2015 | |
|  | Nested | M1-KF | 5’AATGAAGAAGAAATTACTACAAAAGGTGC 3’ | 55 | Abdallah et al., 2015 | |
|  |  | M1-KR | 5 ’GCTTGCATCAGCTGGAGGGCTTGCACCAGA 3’ |  | Abdallah et al., 2015 | |

**Supplementary Table 2 Socio-demographic characteristics of participants (n=354) in the selected districts**

| **Variables** | **Categories** | **Frequency** | **Percent** |
| --- | --- | --- | --- |
| Gender | Female | 197 | 55.6 |
|  | Male | 157 | 44.4 |
| Age Group | 0-14 years | 13 | 3.7 |
|  | 15-24 years | 61 | 17.2 |
|  | 25-47years | 190 | 53.7 |
|  | 48-63years | 61 | 17.3 |
|  | 64years and above | 29 | 8.2 |
| Occupation | Manager or professional level | 29 | 8.2 |
|  | Clerical | 6 | 1.7 |
|  | Sales & Services | 28 | 7.9 |
|  | Skilled Manual | 29 | 8.2 |
|  | Unskilled manual | 183 | 51.7 |
|  | Domestic Services  Others | 79  0 | 22.3  0.0 |
| Residence | urban | 238 | 67.3 |
|  | rural | 116 | 32.7 |
| Dwelling Types | Government supported house | 288 | 81.4 |
|  | Flats | 44 | 12.4 |
|  | Informal settlement | 4 | 1.1 |
|  | Others | 18 | 5.1 |
| Education | No formal education  primary | 0  133 | 0  37.5 |
|  | secondary | 139 | 39.3 |
|  | diploma | 41 | 11.6 |
|  | degree | 24 | 6.8 |
|  | postgraduate | 17 | 4.8 |
| Travel history | inter-district | 229 | 64.6 |
|  | Inter-province | 29 | 8.3 |
|  | Cross border (within Africa) | 6 | 1.8 |
|  | Cross border (outside Africa) | 1 | 0.3 |
|  | No travel history | 89 | 25.0 |

**Supplementary Figure 1. Gel image showing amplified isolates of *Plasmodium spp* and msp-1 genes. MW; 100Bp molecular weight ladder, lanes 1-5, 7, 9; negative isolates for *Plasmodium spp,* lanes 6, 8, 10-13; positive isolates for *Plasmodium spp*, lanes 15-18; amplified *msp-1* genes isolates.**


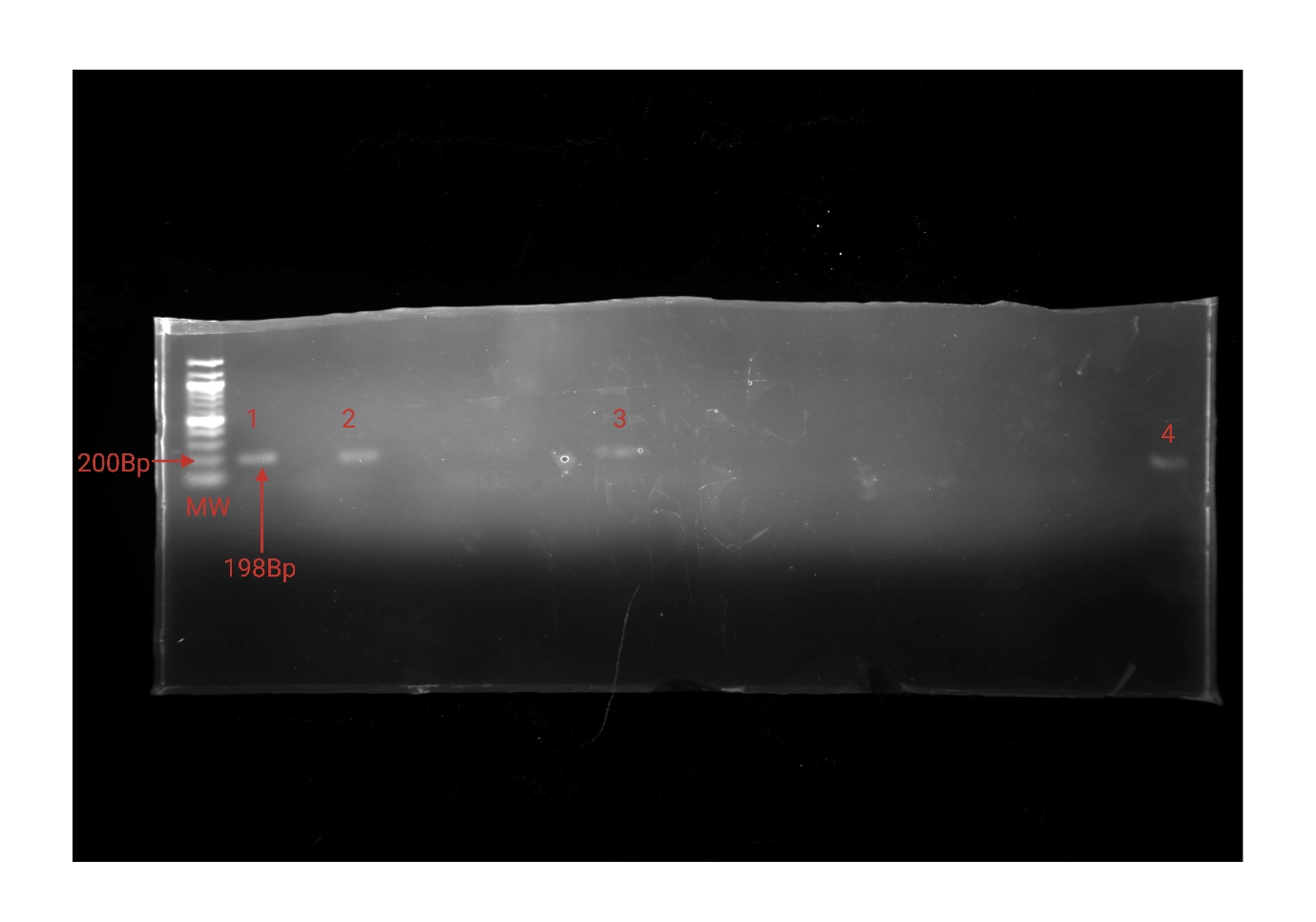


**Supplementary Figure 2. Gel image of amplified MAL7P1. 228 isolates. MW; 100Bp molecular weight ladder; lane 1; Positive control, lanes 2, 3, 4; amplified MAL7P1.228 isolates.**


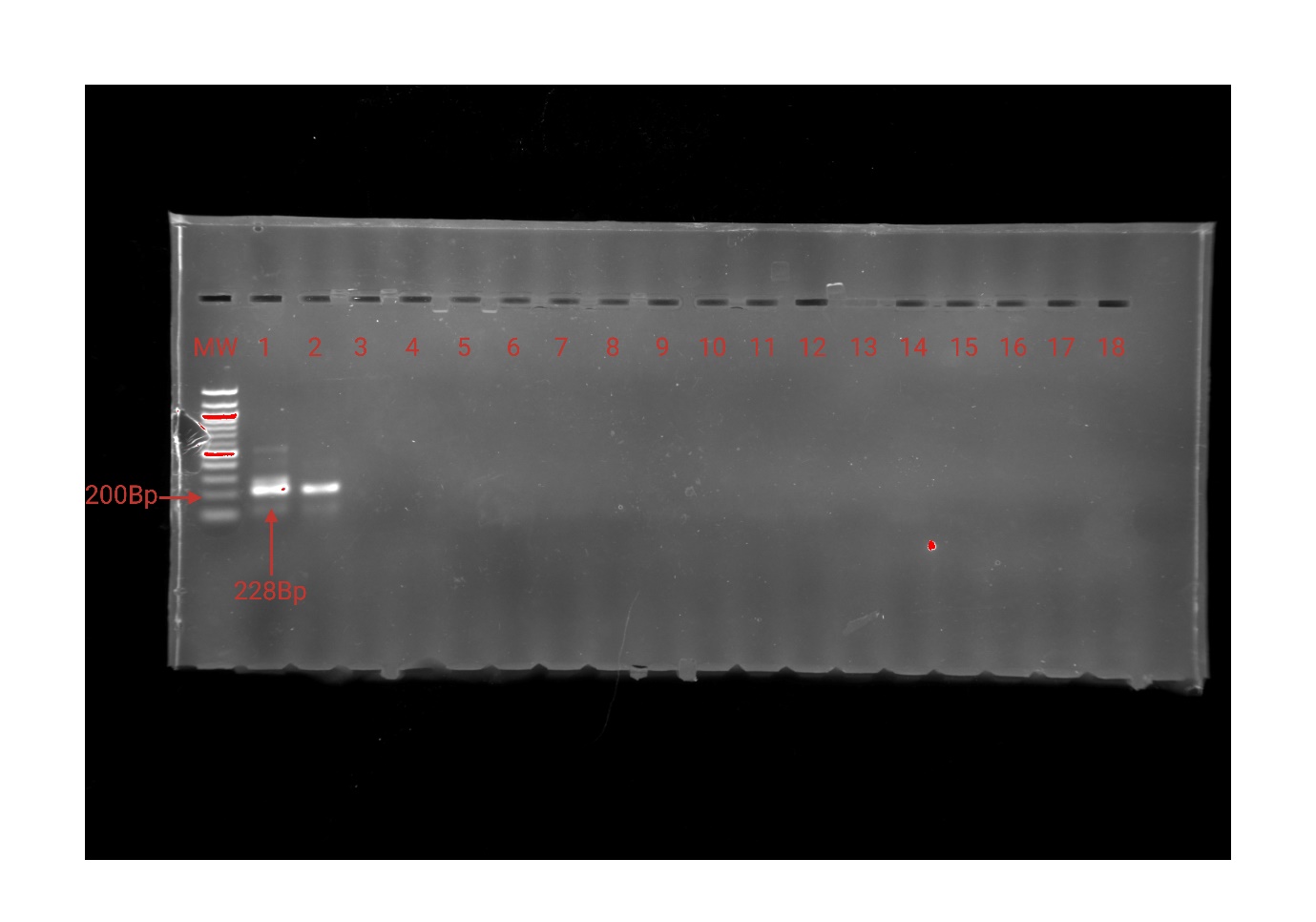


**Supplementary Figure 3. Gel image showing amplified *Pfhrp2* exon. MW; 100Bp molecular weight ladder, lane 1; positive control for *Pfhrp2*, lane 2; amplified isolate, lanes 3-18; negative non-amplifies isolates.**


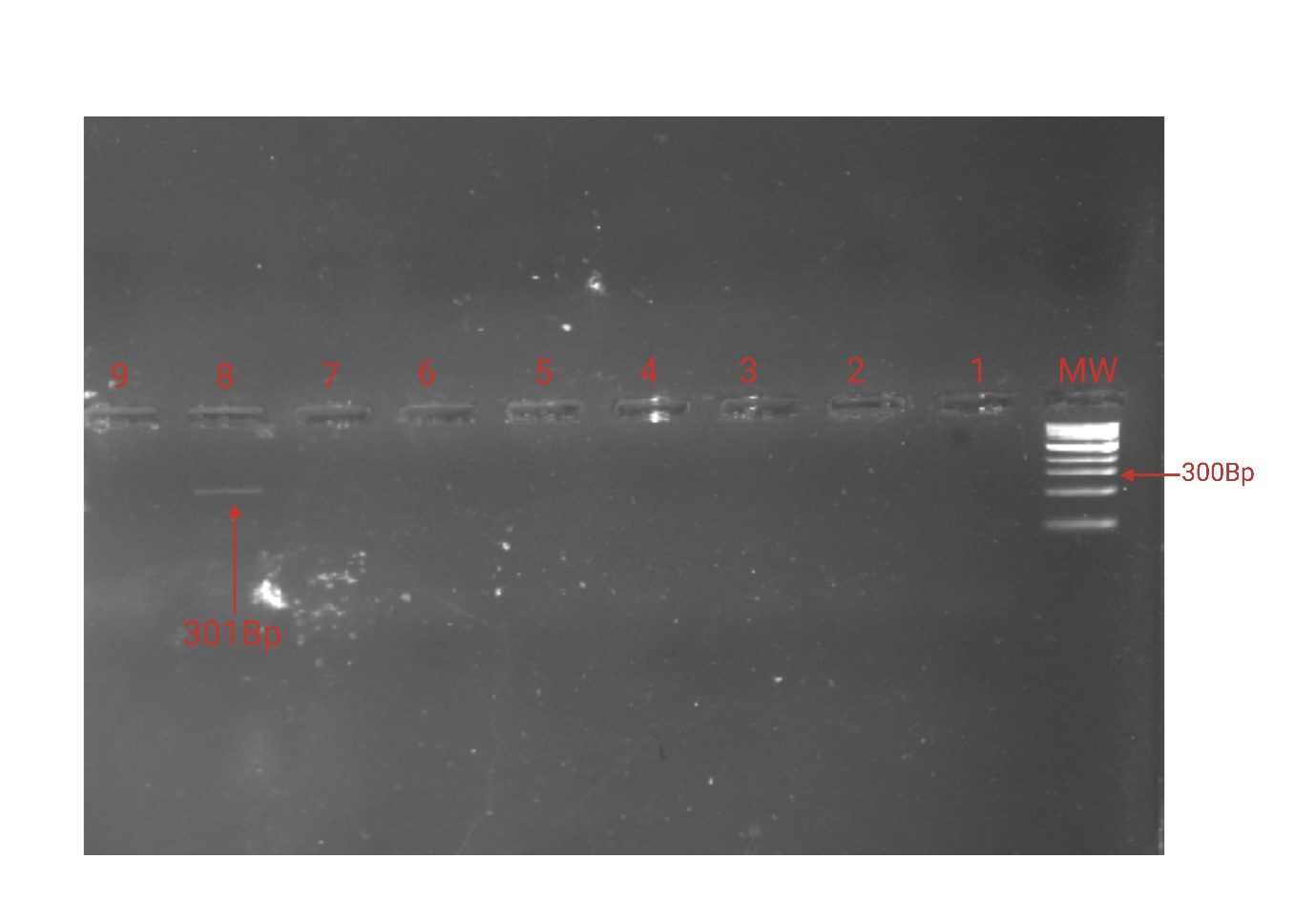


**Supplementary Figure 4. Gel image showing amplified MAL7P1.230 isolate. MW; 100Bp molecular weight ladder, lane 8; amplified isolate of MAL7P1.230, lanes 1-7, 9; negative isolates for MAL7O1.230.**


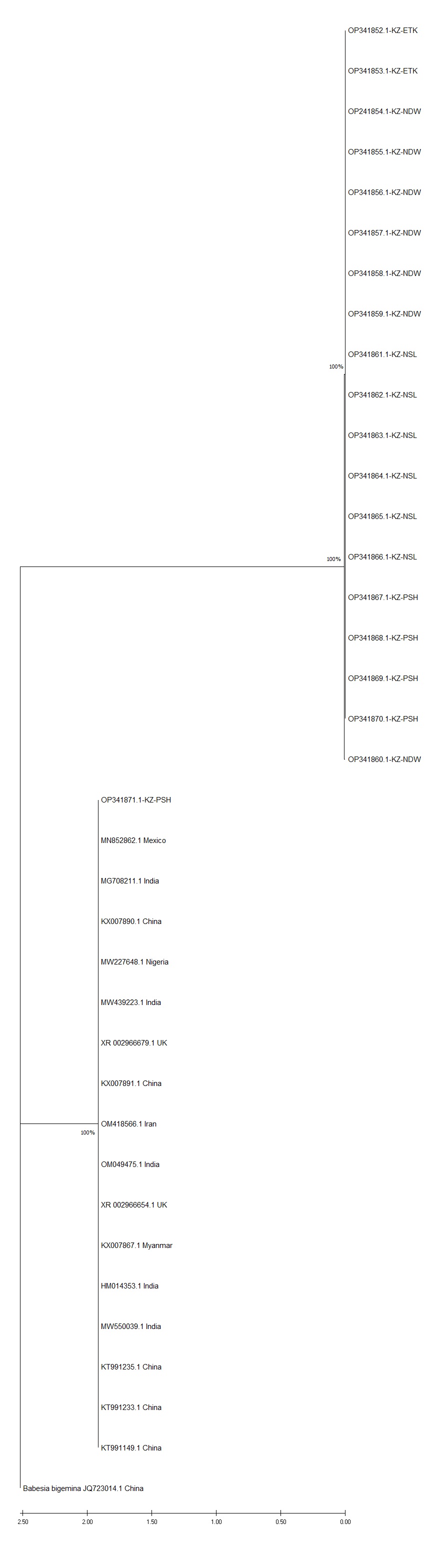


**Supplementary Figure 5 Maximum-Likelihood tree of *18S rRNA* of *Plasmodium falciparum* with bootstraps (1000 replicate) values as indicated.**
